# Purtscher's-Like Retinopathy in Alcoholic Pancreatitis: A Case Report Describing Vision Recovery and Residual Deficits

**DOI:** 10.7759/cureus.96466

**Published:** 2025-11-10

**Authors:** Jessica Holmes

**Affiliations:** 1 Medical Education, University of Hull, Hull, GBR; 2 Paediatrics, Hull Royal Infirmary, Humber Health Partnership, Hull, GBR

**Keywords:** alcohol-induced pancreatitis, alcohol withdrawal syndrome, purtscher's-like retinopathy, purtscher's retinopathy, reduced visual acuity

## Abstract

Purtscher's retinopathy (PR) is a rare ischaemic retinopathy associated with systemic disorders and trauma. Few cases have been reported, and prognosis remains unclear. We present a case of Purtscher's-like retinopathy (PLR) secondary to alcoholic pancreatitis and document visual acuity improvement but persistent visual field deficits. A man in his 50s presented to the hospital with vomiting, seizures and severe abdominal pain on the background of alcohol excess. One day after symptom onset, he developed a sudden, painless loss of vision bilaterally. He was diagnosed with acute alcoholic pancreatitis and alcohol withdrawal and treated accordingly; however, his visual symptoms did not resolve. On assessment, his ocular movements were preserved, but he had significantly reduced visual acuity in both eyes. On fundoscopy, he had widespread retinal ischaemia with blot haemorrhages, and a diagnosis of Purtscher's retinopathy was made. Optical coherence tomography (OCT) and fundus fluorescein angiography (FFA) showed retinal oedema and ischaemic patches, consistent with the diagnosis. In the absence of evidence-based treatment options, he was managed conservatively, without treatment. At 12 weeks, his visual acuity had significantly improved, and he reports almost complete resolution of symptoms. At nine months, he remained asymptomatic; however, he had significantly reduced visual fields bilaterally and an increased cup-to-disc ratio. In the literature, there is a lack of high-quality data on prognosis for Purtscher's retinopathy, as well as poorly documented response to treatment, including oral glucocorticoids, as compared to conservative management. This case illustrates spontaneous visual acuity recovery within 12 weeks but persistent visual field deficits and provides valuable data on progression, imaging findings and outcomes for this rare retinopathy.

## Introduction

Purtscher's retinopathy (PR), named after Otmar Purtscher, who first described the condition in 1910, is an occlusive microangiopathy causing retinal ischaemia and haemorrhages. It typically presents as a sudden, painless loss of vision either unilaterally or bilaterally [1]. Whilst classically associated with trauma, Purtscher's retinopathy (PR) can also occur secondary to systemic conditions such as acute pancreatitis, autoimmune disease or renal failure, where it is called Purtscher's-like retinopathy (PLR) [2]. Pathogenesis is thought to involve retinal arteriolar emboli from fat, fibrin or leucocyte aggregates, though the exact mechanism remains unclear [1].

PR is uncommon, and evidence guiding prognosis and management is limited. Visual outcomes vary widely, from minimal recovery to spontaneous improvement. We present a case of PLR following acute alcoholic pancreatitis with marked visual acuity recovery; however, a persistent significant visual field deficit contributes valuable insight into the natural course and potential for recovery in this rare condition.

## Case presentation

A man in his 50s presented with three days of abdominal pain and new-onset vomiting. He reported sudden-onset, painless, blurred vision bilaterally. His past medical history included chronic alcohol misuse, drinking 500-1000 mL of vodka daily for periods of 6-8 weeks and well-controlled asthma.

On admission, he was disoriented due to alcohol withdrawal. He had severe generalised tenderness on abdominal palpation. In the emergency department, he had a witnessed tonic-clonic seizure with tongue biting, which self-terminated without head injury, and this was attributed to alcohol withdrawal.

Blood tests showed a notably raised C-reactive protein and a raised serum amylase (results shown in Table 1). Other laboratory results were in normal ranges, including triglycerides and calcium.

**Table 1:** Notable blood results at initial assessment A raised C-reactive protein (CRP) and serum amylase

Test	Result	Units	Normal range
C-reactive protein	238	mg/L	<5 mg/L
Serum amylase	387	U/L	28-100 U/L

A computed tomography (CT) scan of his abdomen showed fat stranding and pancreatic oedema; no collections or necrosis was observed. There was no evidence of gallstones or biliary pathology on CT. These findings, coupled with a raised amylase, led to a diagnosis of acute pancreatitis secondary to alcohol excess.

His acute pancreatitis was managed with intravenous 0.9% saline, paracetamol, oral liquid morphine and intravenous co-amoxiclav. Although antibiotics are not in line with standard guidance for acute pancreatitis in the absence of necrosis or collection, he was initiated on antibiotics and completed a five-day course. His alcohol withdrawal was managed with a reducing regimen of chlordiazepoxide and liaison with the local alcohol care team. After receiving treatment, his abdominal pain and infection markers improved; however, his blurred vision remained.

He had a full range of eye movements but blurred vision bilaterally. He had no ophthalmoplegia, no double vision and no nystagmus. He reported dark spots on central vision, such that he could not see his face in reflections and reading was impaired, and he described contrast in light as difficult to differentiate. Prior to admission, he had no vision problems.

**Figure 1:**
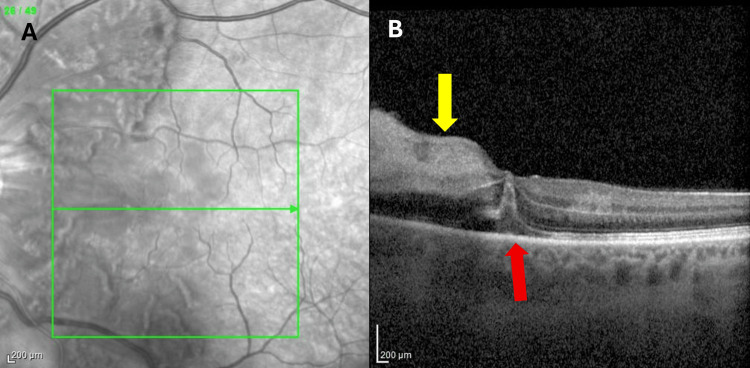
Optical coherence tomography oculus sinister (OS) cut at the level of the fovea on initial assessment Figure 1A shows a face-on view of the fundus demonstrating the location of the corresponding optical coherence tomography (OCT). Figure 1B shows OCT with a hyperreflective lesion in the outer retinal layer consistent with a cotton wool spot and oedema (yellow arrow) and subretinal fluid below the fovea (red arrow)

**Figure 2:**
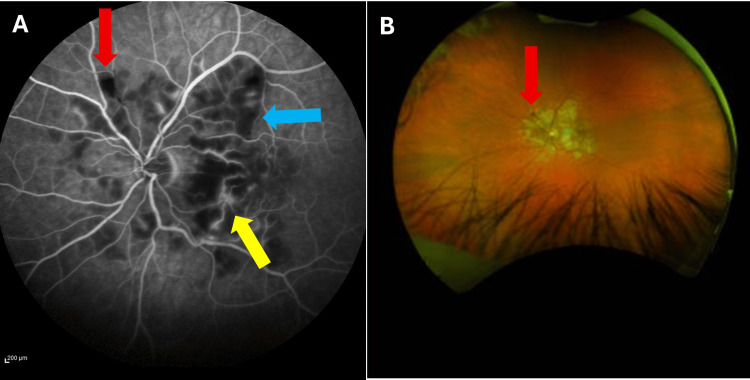
Fundus fluorescein angiography (FFA) and Optos retinal photography oculus sinister (OS) on initial assessment Figure 2A shows oculus sinister fluorescein angiography on initial assessment, late venous phase, and demonstrates the good perfusion of the vascular network, areas of capillary dropout (blue arrow), arteriolar leakage (yellow arrow) and masking due to haemorrhage (red arrow). Figure 2B shows oculus sinister Optos retinal photography on initial assessment, which shows blot haemorrhage (red arrow) corresponding to haemorrhage in Figure 2A

**Figure 3:**
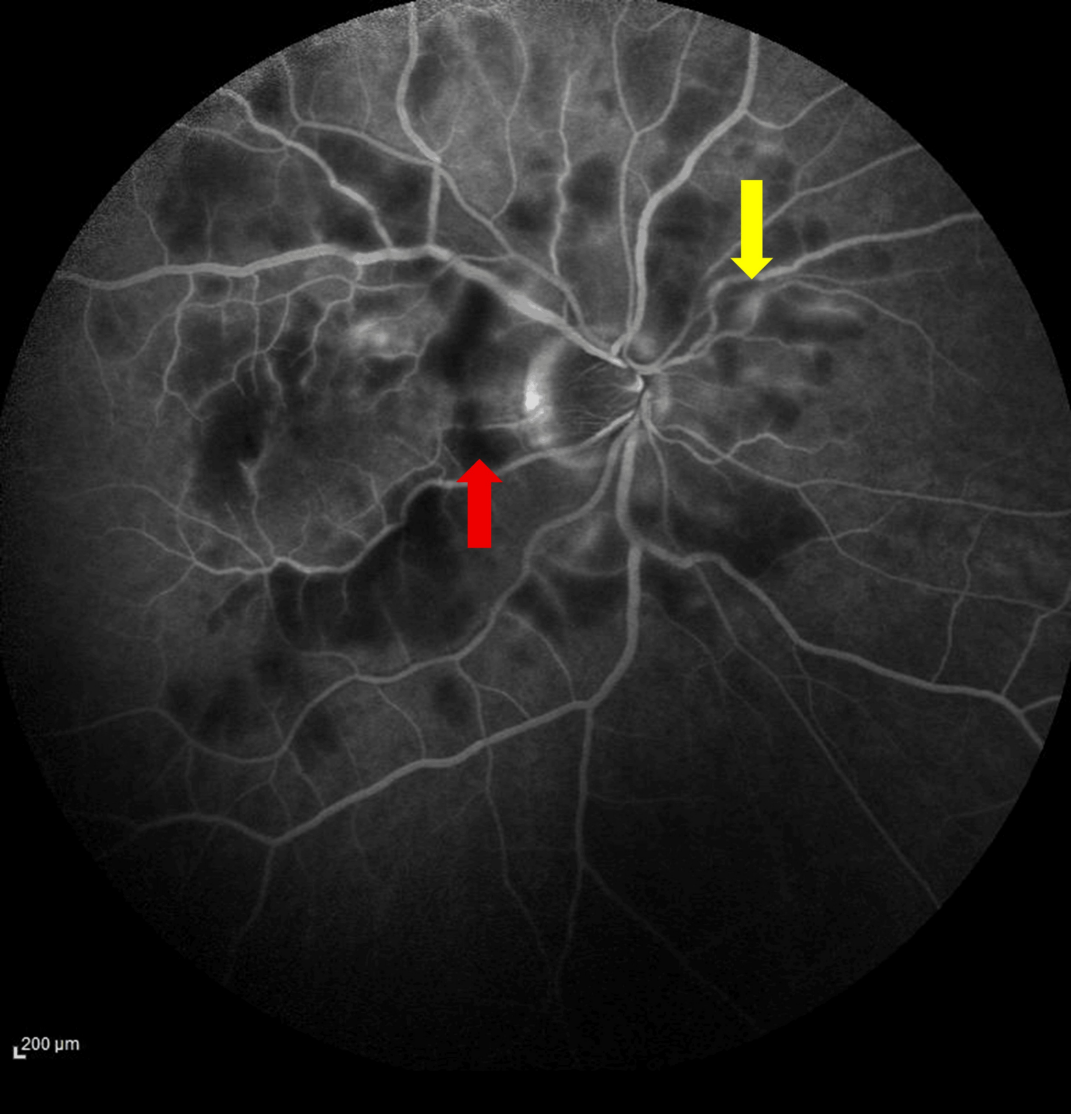
Fluorescein angiography oculus dexter (OD) in the late venous phase on initial assessment The figure demonstrates capillary dropout (red arrow) and hyperfluorescence representing arteriolar leakage (yellow arrow)

Visual acuity on initial examination was 6/60 in the right eye (improving to 6/48 with pinhole correction) and finger counting in the left eye. Bilateral anterior segments were unremarkable. Fundoscopy revealed patchy white areas around the optic discs with blot haemorrhages. Optical coherence tomography (OCT) of the fovea showed retinal oedema bilaterally (Figure 1). Fundus fluorescein angiography (FFA) and Optos (retinal photography) imaging showed bilateral macular oedema, capillary dropout attributable to embolic ischaemia, retinal haemorrhages and cotton wool spots consistent with Purtscher's retinopathy (Figures 2, 3). He was counselled on the guarded prognosis of this rare condition and planned for conservative management with regular follow-up.

One week later, visual acuity improved to 6/24 in the right eye (6/9.5 with pinhole correction) and 6/60 in the left eye (6/19 with pinhole). At 12 weeks, his visual acuity was 6/4.8 on the right and 6/6 on the left. His imaging at 12 weeks was consistent with a previous ischaemic event bilaterally.

**Figure 4:**
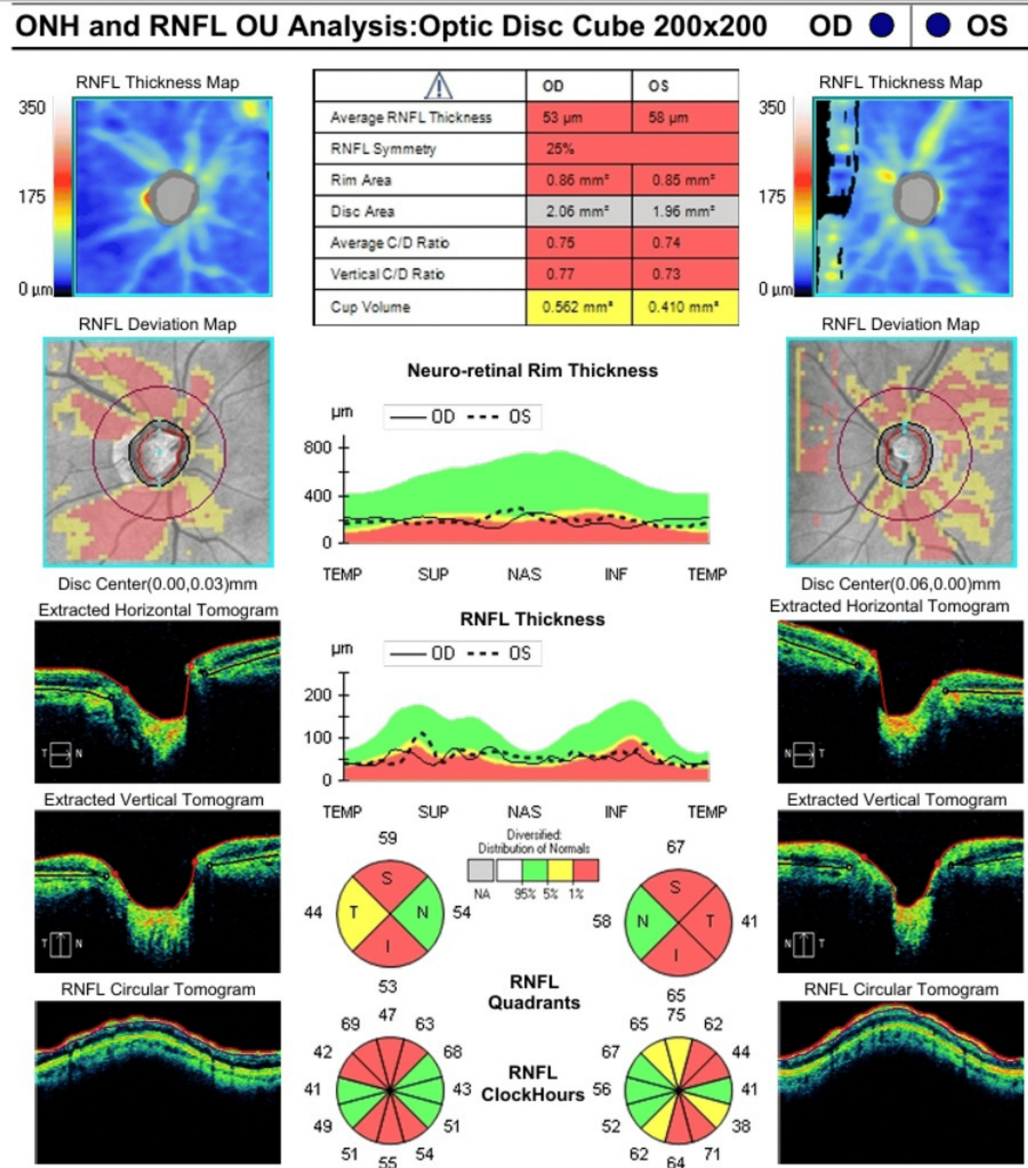
Optical coherence tomography (OCT) of optic disc oculus uterque (OU) at nine months post initial assessment Graphs demonstrate markedly reduced retinal nerve fibre layer (RNFL) thickness with diffuse thinning bilaterally (average RNFL thickness, 53 μm OD and 58 μm OS; normal, >100 μm). There is a significantly enlarged cup/disc (C/D) ratio bilaterally (0.75 OD and 0.74 OS; normal: <0.5), which is indicative of optic nerve loss ONH, optic nerve head; OD, oculus dexter; OS, oculus sinister; TEMP, temporal; SUP, superior; NAS, nasal; INF, inferior

**Figure 5:**
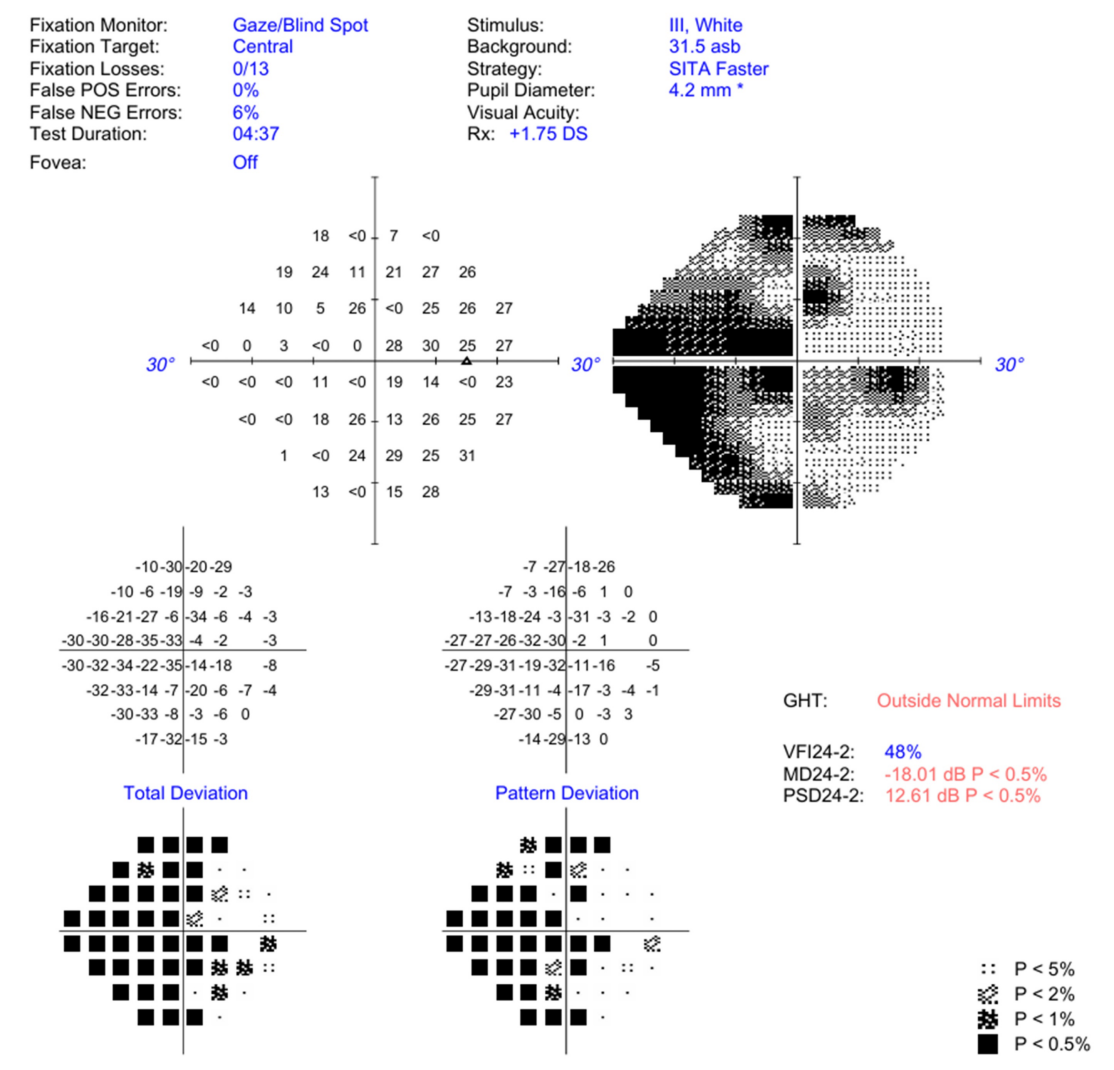
Visual field oculus dexter (OD) at nine months post initial assessment Black areas on grayscale indicate light sensitivity loss, whereas white areas indicate preserved light sensitivity. This graphic shows generalised light sensitivity loss with high focal irregularity. An estimated 48% of the normal visual field function remains *The data for pupil diameter was inputted manually on the visual field analyser GHT, Glaucoma Hemifield Test; VFI24-2, visual field index (24-2 test pattern); MD24-2, mean deviation (24-2 test pattern); PSD24-2, pattern standard deviation (24-2 test pattern); SITA, Swedish Interactive Thresholding Algorithm

At nine months, visual acuity was 6/4.8 on the right and left. OCT of the discs at nine months showed bilateral retinal nerve fibre layer thinning and increased cup-to-disc ratio (Figure 4). Visual field assessment was carried out at nine months, which showed a significant loss in light sensitivity diffusely in both superior and inferior regions (Figure 5). These findings were coupled with normal anterior segments and a normal intraocular pressure. Reduced light sensitivity, retinal nerve fibre layer thinning and increased cup-to-disc ratio are changes consistent with glaucoma. Given these findings, the patient is followed up at regular intervals for the monitoring of intraocular pressure changes and the progression of optic nerve loss.

The patient reported subjective gradual improvement since the initial event. He suffers from cloudy spots on the left field of vision, but these are no longer dark and opaque. He has no ocular pain; however, he reports that he is more sensitive to bright light and colour differentiation is worse than prior to his original admission. This has had minimal impact on his quality of life, and he reports that he is now able to work, drive and cycle, albeit cycle more cautiously. Overall, he describes his recovery as positive, and although his vision is not entirely normalised, he is noticing improvement continually.

## Discussion

Epidemiology and aetiology

Purtscher's retinopathy (PR) is a rare microvascular occlusive disorder, with an estimated incidence of approximately 0.24 per million [3]. Whilst some reviews describe equal incidence in men and women, other authors have identified a higher incidence in men, which may be attributable to higher rates of trauma and acute pancreatitis in the male population [3-5].

Classically, PR occurs following trauma such as in cardiopulmonary resuscitation, long bone fractures or road traffic accidents. When associated with systemic disorders, the term Purtscher's-like retinopathy (PLR) is used. Conditions associated with PLR include renal failure, acute pancreatitis, lupus, fat embolus with acute pancreatitis and systemic lupus erythematosus (SLE), more frequently implicated in PLR [3,5,6].

The pathogenesis is not fully characterised; however, it is thought to be due to microembolic mechanisms. In acute pancreatitis, as in our case, lipase release can cause fat emboli and inflammatory activation leading to aggregates of immune complexes and the occlusion of arterioles in the retina [7]. This results in ischaemia and the characteristic fundoscopy findings.

Presentation and diagnosis

PR and PLR present with acute-onset blurred vision, unilaterally or bilaterally, and reduced visual acuity on assessment [3,4]. Symptoms start 24-48 hours after the precipitating insult. In our case, symptoms fell within this timeframe; however, recognition was delayed as symptoms were attributed to alcohol withdrawal. Similar diagnostic delays in the case of alcohol-induced pancreatitis have been described, highlighting the importance of considering PLR as a differential in patients with vision loss in the context of pancreatitis [8].

Diagnostic criteria proposed in a 2013 systematic review recommended the presence of three or more of the following: Purtscher's flecken, retinal haemorrhages (in low to moderate number), cotton wool spots and complementary investigations compatible with diagnosis and probable cause [5]. Purtscher's flecken are polygonal areas of retinal whitening, similar to cotton wool spots but distinguished by their defined borders. Additional findings on imaging include macular oedema, retinal haemorrhages and a 'pseudo-cherry red spot' similar to that of central retinal artery occlusion [7].

Management and prognosis

Purtscher's retinopathy remains a rare and poorly described condition. There is a lack of consensus on optimal management or prognostic indicators for those with Purtscher's retinopathy.

A recent 2024 review found no significant difference in recovery between different aetiologies of PR, highlighting that outcomes are not dependent on underlying cause [4]. Treatment strategies described in the review broadly fall into one of three categories: conservative management, corticosteroid therapy or anti-vascular endothelial growth factor (VEGF) therapy; also, authors emphasised that due to the lack of data, conclusions on optimal treatment are limited [4].

Systemic corticosteroids have been proposed to reduce inflammation, reduce oedema, stabilise cellular membranes and promote neuronal recovery in partially damaged fibres. Some case studies have shown good results with systemic corticosteroids; however, a systematic review found no significant difference in visual acuity recovery outcomes [9]. As a result, management remains largely supportive. In our case, management was supportive, without the addition of corticosteroid therapy.

In the limited literature, most cases show the spontaneous resolution of symptoms and eye signs [7]. Outcomes are generally reported as best-corrected visual acuity (BCVA) improvement and the resolution of fundoscopy signs. Long-term visual outcomes are not well-reported, with few cases documenting late complications. In our patient, marked BCVA recovery was accompanied by a significant visual field deficit and optic nerve loss, in the absence of raised intraocular pressure. These findings are consistent with normal tension glaucoma; however, it is unclear whether this is secondary to Purtscher's-like retinopathy or independent of precipitating injury. Normal tension glaucoma requires close monitoring for the progression of the disease and can ultimately lead to irreversible sight loss. Therefore, these findings invite further inquiry into the effects of Purtscher's retinopathy on other visual parameters beyond BCVA and the need for extended follow-up.

## Conclusions

Our case illustrates a classic presentation of Purtscher's-like retinopathy secondary to acute alcohol-induced pancreatitis. The patient was managed conservatively and had excellent recovery of visual acuity; however, at nine months, he had significant visual field deficits and optic nerve loss. This case emphasises the importance of maintaining a high index of suspicion when evaluating visual complaints in the context of alcohol-induced pancreatitis, as alcohol intoxication or withdrawal symptoms may mask underlying pathology. Early referral for ophthalmology assessment is crucial to establish a diagnosis and facilitate appropriate follow-up.

The long-term consequences of Purtscher's and Purtscher's-like retinopathy on vision, including the increased risk of normal tension glaucoma, have yet to be characterised or reported in other cases. Given the possible progression to irreversible sight loss in normal tension glaucoma, the need for extended follow-up in Purtscher's retinopathy needs to be explored. This case aims to expand the limited literature that exists on recovery and follow-up of Purtscher's and Purtscher's-like retinopathy.
